# Effect of Baduanjin exercise in a blended online-offline model on cognitive function and peripheral blood BDNF levels in older adults

**DOI:** 10.3389/fphys.2025.1573674

**Published:** 2025-06-18

**Authors:** Zhihui Zhou, Jiawei Wang, Fanhui Kong, Qianqian Zhang

**Affiliations:** ^1^ School of Athletic Performance, Shanghai University of Sport, Shanghai, China; ^2^ College of P.E. and Sports, Beijing Normal University, Beijing, China; ^3^ Faculty of Physical Education, Fudan University, Shanghai, China; ^4^ Department of Physical Education, Shanghai Jiao Tong University, Shanghai, China

**Keywords:** Baduanjin, online-offline model, cognitive function, BDNF, older adults

## Abstract

**Objective:**

This study aimed to examine the effect of 12-week Baduanjin exercise on cognitive function and peripheral blood brain-derived neurotrophic factor (BDNF) levels in the older adults, utilizing a blended online and offline model.

**Methods:**

A total of 40 older adults with low physical activity levels were randomly assigned to either Baduanjin group (BG, n = 20, age: 63.75 ± 1.41 years) or control group (CG, n = 20, age: 63.10 ± 1.65 years). The BG participated in 12-week Baduanjin exercise, conducted 3 times per week, integrating both online and offline components. Each session comprised a 60-min structure, including a 10-min warm-up, 40 min of Baduanjin exercise, and a 10-min cool-down period. Cognitive function and peripheral blood BDNF levels were assessed prior to and following the 12-week intervention.

**Results:**

(1) After 12-week intervention, BG exhibited significant improvements in MoCA (*P* < 0.01), and MoCA of BG was significantly higher than CG (*P* < 0.05). (2) After 12-week intervention, BG exhibited significant improvements in DST-F (*P* < 0.05), DST-B (*P* < 0.05), VFT (*P* < 0.01), Stroop-A (*P* < 0.05), and Stroop-RT (*P* < 0.05). DST-F, DST-B, VFT, and Stroop-A scores of BG were significantly higher than those of the CG (*P* < 0.05) after intervention. And Stroop-RT was significantly lower than CG (*P*<0.05) after intervention. (3) BDNF levels: After 12-week intervention, BG exhibited significant improvements in BDNF (*P* < 0.01), and BDNF of BG was significantly higher than CG (*P* < 0.05). (4) A significant positive linear correlation was identified between MoCA scores and BDNF levels (r = 0.488).

**Conclusion:**

12-week Baduanjin intervention in a blended online-offline model can significantly improve cognitive function in the older adults. The improvement in cognitive levels induced by exercise is accompanied by an increase in peripheral blood BDNF content, and there is a significant positive correlation between the two factors.

## 1 Introduction

Aging is invariably accompanied by a gradual decline in various physiological functions among the elderly, with cognitive impairment being one of the most salient and consequential (Simen et al., 2011). Such cognitive decline not only exerts a detrimental effect on the quality of life experienced by the elderly but, when severe, can precipitate neurodegenerative disorders such as dementia, thereby significantly impairing their ability to lead a normal life (Miksys and Tyndale, 2010). Additionally, it places a considerable psychological burden on individuals and their families and entails substantial economic costs for society and the nation. Consequently, identifying effective strategies to mitigate the decline in cognitive function among the elderly has emerged as a pressing issue that demands our attention.

Since the discovery of brain-derived neurotrophic factor (BDNF) in the 1980s (Barde et al., 1982), its pivotal role in promoting hippocampal neurogenesis and enhancing learning and memory functions has been extensively investigated and widely acknowledged (Gottmann et al., 2009). Increasing BDNF levels in the brain to facilitate hippocampal neurogenesis is an important avenue for improving cognitive function. Research indicates that exercise can lead to increased BDNF expression in the brain and promote hippocampal neurogenesis (Lei et al., 2019). Individuals in middle and old age who regularly engage in physical activities exhibit a reduced risk of overall cognitive decline (Weuve et al., 2004) and a lower incidence of dementia (Abbott et al., 2004).

Research has indicated that exercise improves cerebral blood flow perfusion and vascular endothelial function, enhancing oxygenation levels in key brain regions such as the prefrontal cortex, thereby optimizing neural network efficiency (Li et al., 2024). Long-term regular exercise increases hippocampal gray matter volume by approximately 2% and enhances myelination of white matter fiber tracts, delaying age-related brain atrophy (Han et al., 2024). At the molecular level, exercise activates the PGC-1α pathway to boost mitochondrial biogenesis and enhance neuronal energy metabolism efficiency. Concurrently, epigenetic regulation mechanisms (e.g., histone acetylation) upregulate the expression of learning- and memory-related genes, such as *c-Fos* (Li et al., 2024). Therefore, appropriate and regular physical exercise can effectively delay the decline of cognitive function in the brain, enhance learning and memory capabilities, and particularly exert a positive influence on certain neurodegenerative diseases, such as Alzheimer’s disease (Ahlskog et al., 2011; Fang et al., 2017).

The intensity and form of exercise are two important factors that have been extensively explored (Knaepen et al., 2010; Brown et al., 2012). Baduanjin, a traditional form of exercise in China, differs from other forms of exercise in that it emphasizes the coordination between the body and mind of the practitioner. Studies have shown that Baduanjin exercise can slow down the normal age-related decline in memory domains and is more effective than jogging (Sun and Wang, 2008). However, there is currently a paucity of research on the effects of Baduanjin on the cognitive function of the elderly, and further investigation is needed to explore its impact on cognitive function in this population. Moreover, traditional exercise interventions primarily rely on face-to-face interactions, which may result in participant dropout due to travel constraints, time limitations, and associated costs. Smart phone applications such as WeChat and Tencent Meeting can facilitate real-time, face-to-face video teaching online, providing remote monitoring, feedback, and interaction with others, which may be crucial for promoting long-term exercise adherence and sustained functional improvement among participants. Therefore, this study investigates the effects of Baduanjin exercise on cognitive function and peripheral blood BDNF levels in the older adults through a blended online and offline mode.

## 2 Methods

### 2.1 Participants

Forty older adults with low levels of physical activity, who met the inclusion criteria, were recruited from the community and randomly assigned to Baduanjin exercise group (BG, n = 20) or a control group (CG, n = 20), using a random number generator. The CG continued their usual daily physical activities without engaging in any additional exercise programs, while BG participated in a 12-week Baduanjin exercise program. During the intervention period, participants were instructed to maintain their existing lifestyle and dietary habits. All participants provided written informed consent, and the study was approved by the Ethics Committee of Beijing Sport University. The basic demographic information of the participants is presented in Table 1. Inclusion Criteria: (1) Older adults aged 60–75 years, regardless of gender. (2) No history of alcohol abuse or smoking addiction. (3) No severe coronary heart disease, diabetes, hypertension, or neurodegenerative diseases. (4) Not participating in any other form of experimental intervention organized by any entity during the study period. (5) Reported low levels of physical activity as assessed by the International Physical Activity Questionnaire (IPAQ) (Macfarlane et al., 2011).

**TABLE 1:** Basic information.

Group	N	Age (years)	BMI (kg/m^2^)	Education year (years)	IPAQ (METs/week)	MoCA score
BG	20	63.75 ± 1.41	25.60 ± 0.58	10.45 ± 1.23	430 ± 70.35	26.20 ± 0.14
CG	20	63.10 ± 1.65	25.34 ± 0.64	10.20 ± 1.28	426 ± 74.42	26.55 ± 0.15

BMI, body mass index; IPAQ, international physical activity questionnaire. Values are mean ± SD.

### 2.2 Exercise intervention

**FIGURE 1:**
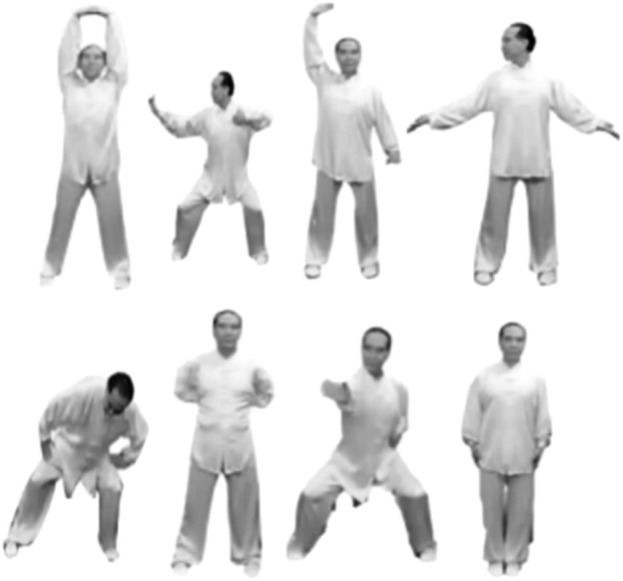
Baduanjin postures.

The Baduanjin exercise was employed as the intervention for the participants in BG. The entire intervention process was divided into two phases. Phase 1: “Foundation Building Phase.” This phase involved five sessions per week of offline instruction and correction of Baduanjin movements. Professional instructors conducted theoretical lectures, answered questions, and corrected movements with each session lasting 60 min. Phase 2: “Intervention Implementation Phase.” This phase consisted of a 12-week exercise intervention with heart rate monitoring. The first 4 weeks were conducted offline, while the remaining 8 weeks were facilitated through WeChat for online instruction. Participants were required to exercise three times per week, with each session lasting 60 min, comprising 10 min of warm-up, 40 min of Baduanjin exercise, and 10 min of cool-down. Each training session was guided by a professional coach with 10 years of experience in Baduanjin exercise. Attendance and exercise details of the participants were recorded. The Baduanjin training protocol consisted of eight postures, as illustrated in the Figure 1.

### 2.3 Measurements

Pre- and post-tests were conducted after the completion of the exercise intervention. Participants were instructed to refrain from smoking, consuming strong tea, coffee, and alcohol for 12 h prior to the testing. All testing procedures were conducted in an environment with a temperature of 24°C–26°C, and both pre- and post-tests were completed by the same tester during the same time period.

#### 2.3.1 Overall cognitive function

The Montreal Cognitive Assessment (MoCA) was administered to all participants before and after the intervention to assess their Overall Cognitive Function (Chen et al., 2016). The total score ranges from 0 to 30, with higher scores indicating better cognitive performance.

#### 2.3.2 Specific domain cognitive function

The Digit Span Test (DST) was administered to all participants before and after the intervention to assess short-term memory capacity in the cognitive function of the elderly (Lezak et al., 2012). Participants were presented with a sequence of digits, with each digit displayed for 1 s. After the entire sequence was shown, participants were required to repeat the digits in order or in reverse order. Record the scores of Digit Span Test - Forward (DST-F) and Digit Span Test - Backward (DST-B).

The Verbal Fluency Test (VFT) was conducted on all participants before and after the intervention to evaluate language ability and semantic memory (Mok et al., 2004). Participants were asked to name as many words as possible within a specified category within a 1-min time frame in vessel diameter. FMD (%) = (maximum diameter -basal diameter) ÷ basal inner diameter ×100%.

The Stroop Color-Word Task (SCWT) was performed on all participants before and after the intervention using E-Prime 2.0 software to assess executive function in the cognitive abilities of the elderly (Scarpina and Tagini, 2017). Participants were tasked with quickly and accurately identifying and judging the colors of the Chinese characters “red,” “green,” “yellow,” and “blue” as they appeared sequentially in the center of the screen. Participants were instructed to ignore the pronunciation or meaning of the characters and focus on assessing and rapidly determining the color of the characters. Record Stroop Color-Word Task reaction time (Stroop-RT) and accuracy (Stroop-A).

#### 2.3.3 Blood marker tests

Blood samples were collected once before and once after the intervention. Participants were required to fast for 12 h, refrain from smoking, and avoid consuming strong tea, coffee, and alcohol. Blood samples (10 mL of fasting venous blood) were drawn between 7:00 and 8:00 a.m. The samples were allowed to stand at room temperature for 1 h and then centrifuged at 4,000 rpm for 10 min. The serum was extracted and stored in an ultra-low temperature freezer at −80°C. The concentration of serum BDNF was measured using the enzyme-linked immunosorbent assay (ELISA) method.

### 2.4 Statistics and analysis

The statistical analysis of the experimental data was conducted using SPSS23.0. The Shapiro-Wilk (S-W) test was employed to assess the normality of the data. A two-way repeated measures analysis of variance (ANOVA) was utilized to compare the data obtained after the Baduanjin exercise intervention, both within and between groups. The between-subjects factor was group (intervention group, control group), and the within-subjects factor was testing time (pre-test, post-test). Pearson’s correlation coefficient was used to assess the correlation between relevant indicators. Descriptive statistics are presented as mean ± standard deviation (Mean ± SD). Statistical significance was set at *P* < 0.05, with high significance at *P* < 0.01.

## 3 Results

### 3.1 Effect of Baduanjin on overall cognitive function in the older adults

**FIGURE 2:**
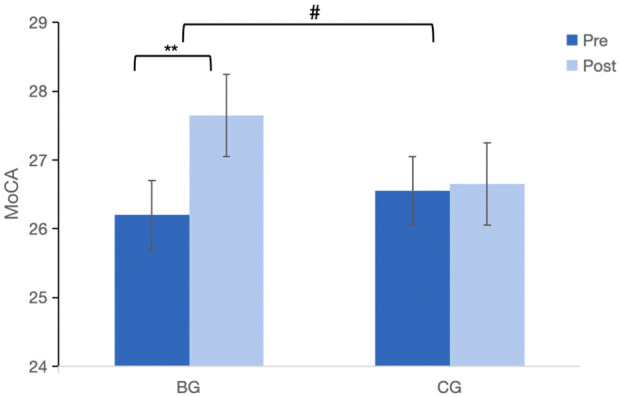
Effect of Baduanjin on MoCA in the older adults. MoCA, montreal cognitive assessment; Values are mean ± SD. **p < 0.01, intra-group comparison; #*p* < 0.05, represents the comparison between BG and CG in post-intervention.

As shown in Figure 2, no significant difference in MoCA scores between BG and CG at baseline (*p* > 0.05). There was a significant Time × Group interaction (F = 37.24, *p* < 0.05, partial η2 = 0.47), indicating that the BG improved more than the CG after 12-week intervention. The BG showed significant improvement in MoCA scores after the intervention (*p* < 0.05), while the CG did not (*p* > 0.05). Post-intervention, the BG had higher MoCA scores than the CG (*p* < 0.05).

### 3.2 Effect of Baduanjin on specific domain cognitive function in the older adults

**FIGURE 3:**
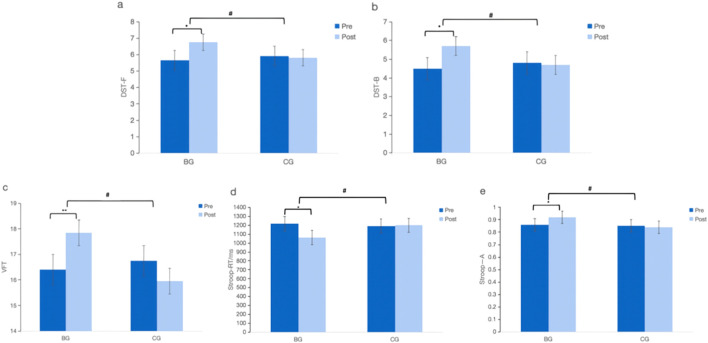
Effect of Baduanjin on Specific Domain Cognitive Function in the older adults. **(a)**: DST-F, digit span test-forward; **(b)**: DST-B, digit span test-backward; **(c)**: VFT, verbal fluency test; **(d)**: Stroop-RT; stroop color-word task reaction time; **(e)**: Stroop-A, stroop color-word task accuracy.

As shown in Figure 3, no significant difference in DST-F, DST-B, VFT, Stroop-A scores, Stroop-RT between BG and CG at baseline (*p* > 0.05). There was a significant Time × Group interaction in DST-F (F = 27.94, *p* < 0.05, partial η2 = 0.42), DST-B (F = 35.37, *p* < 0.05, partial η2 = 0.48), VFT (F = 6.65, *p* < 0.05, partial η2 = 0.48), Stroop-A (F = 4.21, *p* < 0.05, partial η2 = 0.59) and Stroop-RT (F = 76.04, *p* < 0.05, partial η2 = 0.67) indicating that the BG improved more than the CG after 12-week intervention. The BG showed significant improvement in DST-F (*p* < 0.05), DST-B (*p* < 0.05), VFT (*p* < 0.01), Stroop-A (*p* < 0.05), and Stroop-RT (*p* < 0.05) scores after the intervention, while the CG did not (*p* > 0.05). Post-intervention, the BG had higher DST-F, DST-B, VFT, Stroop-A and Stroop-RT scores than the CG (*p* < 0.05).

### 3.3 Effect of Baduanjin on BDNF levels in the older adults

**FIGURE 4:**
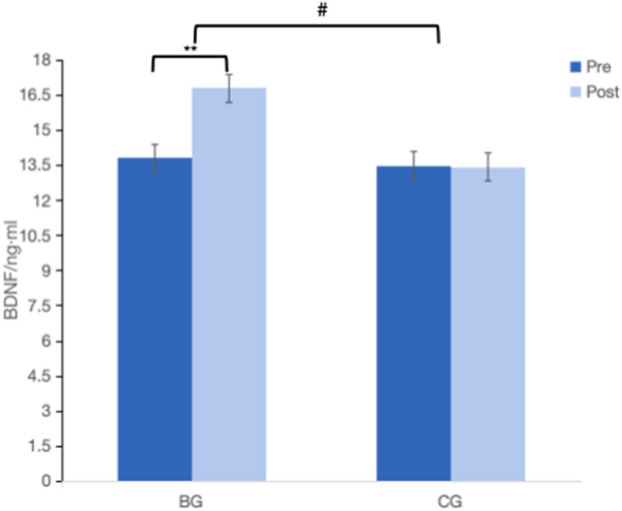
Effect of Baduanjin on BDNF in the older adults. BDNF, brain-derived neurotrophic factor. Values are mean ± SD. **p < 0.01, intra-group comparison; #*p* < 0.05, represents the comparison between BG and CG in post-intervention.

As shown in Figure 4, no significant difference in BDNF levels between BG and CG at baseline (*p* > 0.05). There was a significant Time × Group interaction (F = 52.95, *p* < 0.05, partial η2 = 0.59), indicating that the BG improved more than the CG after 12-week intervention. The BG showed significant improvement in BDNF levels after the intervention (*p* < 0.01), while the CG did not (*p* > 0.05). Post-intervention, the BG had higher BDNF levels than the CG (*p* < 0.05).

### 3.4 Correlation analysis between MoCA and BDNF

**FIGURE 5:**
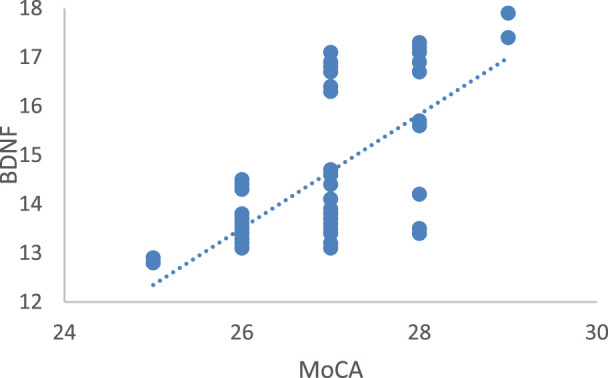
Correlation between MoCA and BDNF. MoCA, montreal cognitive assessment; BDNF, brain-derived neurotrophic factor.

As shown in Figure 5, a linear positive correlation was found between MoCA and BDNF (r = 0.488, *P* = 0.001), indicating that individuals with higher cognitive ability scores also have higher BDNF levels.

## 4 Discussion

This study primarily investigated the effects of 12-week Baduanjin exercise intervention delivered through a combination of online and offline modes on overall cognitive function and specific domain cognitive functions in the older adults, aiming to identify the cognitive components susceptible to exercise-induced changes. This study found MoCA, DST, VFT, Stroop and BDNF levels significantly improved after 12-week Baduanjin exercise.

Cognitive function is the most central and dynamic element of human psychological activities, comprising multiple domains including orientation, attention, memory, calculation, and executive function (Murman, 2015). Extensive research has shown that elderly individuals who regularly engage in physical exercise exhibit better cognitive performance, with the cognitive gap compared to younger individuals narrowing (Chang et al., 2010). Law et al. found that exercises such as Tai Chi and yoga have a positive impact on language and learning cognitive functions (Law et al., 2014). Studies have shown that a 12-week Baduanjin fitness program can significantly enhance the brain gray matter volume in the medial temporal lobe, insula, and putamen of the elderly; improve the resting functional connectivity between the hippocampus and the medial and dorsolateral prefrontal cortex, thereby enhancing cognitive function and effectively slowing down the decline in memory function in the elderly (Tao et al., 2017a; Tao et al., 2017b). The results of this study indicate that a 12-week Baduanjin exercise intervention, delivered through a combination of online and offline modes, significantly improves overall cognitive function, short-term memory capacity, language ability and semantic memory, and executive function in the older adults. Baduanjin exercise integrates mental and physical activities, where the practice of its techniques stretches the body’s muscles, fascia, ligaments, and joint capsules, promoting local blood circulation in the limbs, stimulating the body’s proprioceptive capabilities, and enhancing the nervous system’s ability to recruit muscles (Koh, 1982). This exercise modality is a combination of both aerobic and anaerobic training, with elements of psychological training also involved. Therefore, Baduanjin exercise can effectively delay the decline in cognitive function in the older adults.

Neuroplasticity is an ongoing process that allows for the short-term, medium-term, and long-term reshaping of synaptic organization. As diseases progress, the regenerative capacity and plasticity of hippocampal neurons in individuals with cognitive impairments continuously decline (Kuehn, 2015). BDNF involves in neuronal differentiation, growth, injury repair, and the formation of new synapses, and plays a crucial role in the regulation of brain plasticity and memory function (Petzinger et al., 2013). Exercise mediates the impact of peripheral physical activity on the central nervous system by regulating BDNF levels in the hippocampus of the brain (Cassilhas et al., 2012; Bechara and Kelly, 2013). In other words, exercise is an important non-pharmacological way to regulate BDNF expression in the brain, playing a positive role in promoting learning and memory and improving neurodegenerative diseases (Loprinzi and Frith, 2019). Studies have found that exercise can lead to an increase in BDNF levels in multiple brain regions, primarily in the hippocampus, which is accompanied by hippocampal cell proliferation and improved cognitive function (Liu and Nusslock, 2018). This study measured the serum BDNF levels of two groups of subjects before and after exercise intervention. The results showed that the serum BDNF levels of the older adults after a 12-week Baduanjin intervention, delivered through a combination of online and offline modes, significantly increased compared to pre-intervention levels, and there was also a significant difference compared to the control group. Combining MoCA scores and BDNF levels of correlation analysis results, we can discover a significant positive correlation between the improvement in cognitive function in the elderly and BDNF levels in peripheral blood. These findings are in accordance with prior research correlating peripheral blood BDNF levels with cognitive function in individuals with cognitive impairments. Specifically, peripheral blood BDNF reduced, and cognitive function damaged. After treatment, symptoms ameliorate, and cognitive function and BDNF concentration exhibit concurrent enhancement (MacPherson, 2017). It can be seen that peripheral blood BDNF is one of the key factors reflecting the relationship between exercise and the promotion of cognitive function.

## 5 Conclusion

12-week Blended Online-Offline Model Baduanjin exercise intervention can significantly improve cognitive function in the older adults. Moreover, the improvement in cognitive function induced by exercise is accompanied by an increase in peripheral blood BDNF levels, and there is a significant positive correlation between the two. Peripheral blood BDNF is one of the key factors reflecting the relationship between exercise and the promotion of cognitive function.

## Data Availability

The raw data supporting the conclusions of this article will be made available by the authors, without undue reservation.
